# Left Main Coronary Artery Compression following Melody Pulmonary Valve Implantation: Use of Impella Support as Rescue Therapy and Perioperative Challenges with ECMO

**DOI:** 10.1155/2014/959704

**Published:** 2014-03-18

**Authors:** Erica D. Wittwer, Juan N. Pulido, Shane M. Gillespie, Frank Cetta, Joseph A. Dearani

**Affiliations:** ^1^Division of Critical Care Medicine and Cardiothoracic Anesthesia, Department of Anesthesiology, Mayo Clinic College of Medicine, 200 First Street SW, Rochester, MN 55905, USA; ^2^Division of Pediatric Cardiology, Department of Pediatrics, Mayo Clinic, Rochester, USA; ^3^Division of Cardiovascular Surgery, Department of Surgery, Mayo Clinic, Rochester, USA

## Abstract

The purpose of this case is to describe the complex perioperative management of a 30-year-old woman with congenital heart disease and multiple resternotomies presenting with pulmonary homograft dysfunction and evaluation for percutaneous pulmonary valve replacement. Transvenous, transcatheter Melody valve placement caused left main coronary artery occlusion and cardiogenic shock. An Impella ventricular assist device (VAD) provided rescue therapy during operating room transport for valve removal and pulmonary homograft replacement. ECMO support was required following surgery. Several days later during an attempted ECMO wean, her hemodynamics deteriorated abruptly. Transesophageal and epicardial echocardiography identified pulmonary graft obstruction, requiring homograft revision due to large thrombosis. This case illustrates a role for Impella VAD as bridge to definitive procedure after left coronary occlusion and describes management of complex perioperative ECMO support challenges.

## 1. Case Report

**Figure 1:**
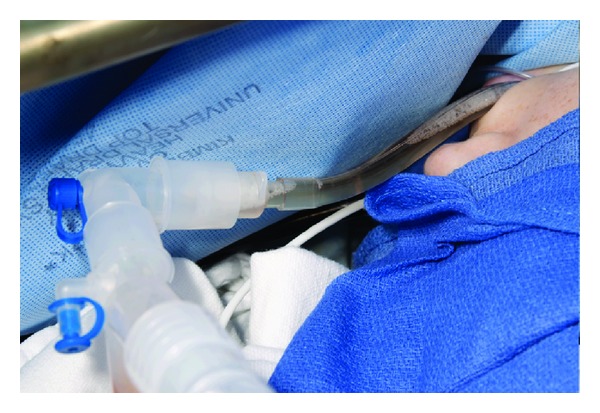
Intraoperative photo demonstrating severe pulmonary edema following Melody valve deployment with development of cardiogenic shock. Note complete filling of endotracheal tube with pulmonary edema fluid.

**Figure 2:**
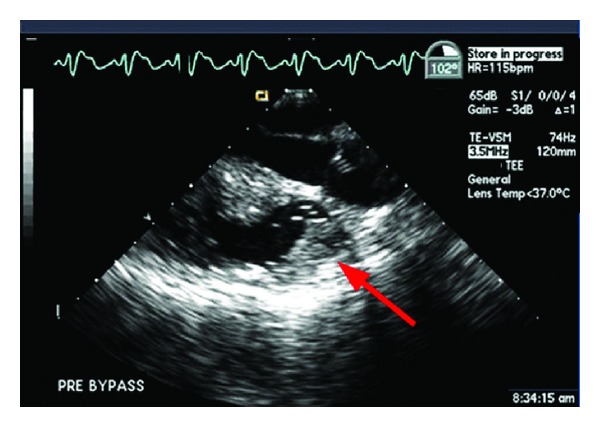
Transesophageal echocardiography: midesophageal aortic valve long axis view at 102°. Evidence of obstruction in the right ventricular outflow tract (Arrow) and pulmonary conduit.

**Figure 3:**
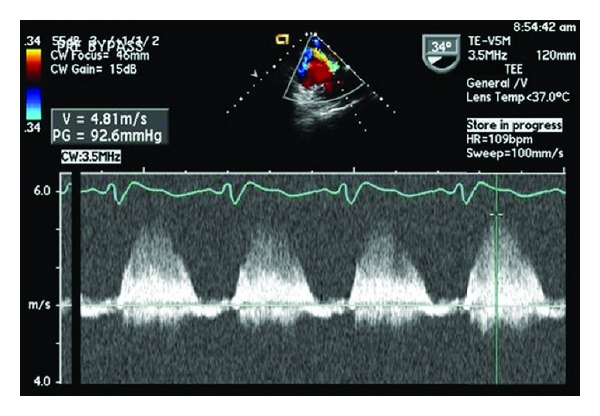
Transesophageal echocardiography: midesophageal 4 chamber view with emphasis in tricuspid valve at 34°. Continuous wave Doppler assessment of tricuspid valve regurgitation used to calculate right ventricular systolic pressure (RVSP). Note peak pressure gradient of 92 mm Hg.

**Figure 4:**
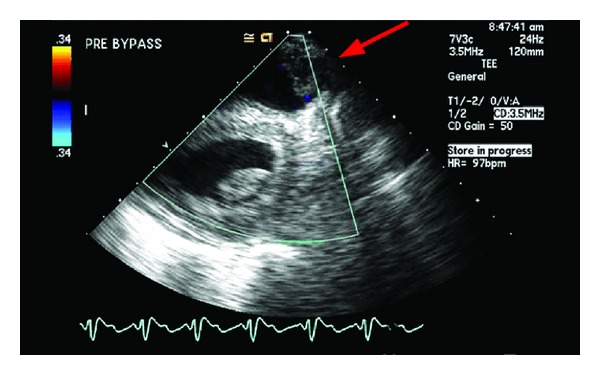
Intraoperative epicardial ultrasound imaging of the right ventricular outflow tract (RVOT) and pulmonary conduit. Arrow: filling defect obstructing the RVOT.

A 30-year-old woman with prior Ross procedure for subaortic stenosis, presented with moderate to severe pulmonary homograft stenosis/regurgitation and New York Heart Association class II symptoms. Due to four prior sternotomies, she was considered high surgical risk and the decision was made to proceed with percutaneous transvenous transcatheter pulmonary valve replacement (Melody valve (Medtronic Inc., Minneapolis, MN, USA)) in the catheterization laboratory. After initial balloon inflation, there was visible homograft enlargement, and simultaneous aortic root angiogram was performed demonstrating patent right and left coronary arteries (LCA). The right ventricular outflow tract was then prepared with successful placement of two Palmaz stents. Repeat coronary angiography demonstrated patent LCA prior to Melody valve deployment. The Melody valve was then deployed and the patient developed progressive, severe hypotension, bradycardia, and cardiogenic shock. Transthoracic echocardiogram (TTE) demonstrated akinesis of the lateral and anterior walls of the left ventricle (LV) and severe acute mitral valve regurgitation. She instantly developed severe pulmonary edema with fluid filling the endotracheal tube and anesthesia circuit (Figure 1). A repeat coronary angiogram demonstrated acute LCA occlusion. CPR was instituted in an attempt to crush the valve and relieve the obstruction without success or hemodynamic improvement. Arrangements were immediately made for transport to the operating room, but the patient needed cardiovascular support in the interim. An Impella 2.5 percutaneous left ventricular assist device (LVAD) was placed via the femoral artery to assist with cardiac output and LV decompression while the patient was transported to the operating room for definitive therapy. Femoral-femoral cardiopulmonary bypass (CPB) was instituted and subsequently proceeded with fifth time sternotomy, Melody valve, and pulmonary homograft explantation and right ventricular outflow tract (RVOT) reconstruction with a 22 mm Contegra graft. After CPB, she remained in cardiogenic shock requiring high dose inotropic and vasopressor support. She had recurrent severe acute pulmonary edema and hypoxemic respiratory failure despite high dose inhaled alprostadil (160 mcg/hr), 100% FiO_2_, and PEEP of 10 cm H_2_O. Venoarterial ECMO was then instituted via femoral vessels and she was transported to the ICU in critical condition. On the fourth day of ECMO support, she developed a large hemothorax requiring chest tube decompression, six units of RBCs, and two units of platelets. The heparin infusion was subsequently held and bleeding resolved over several hours. The following day, the patient was requiring minimal inotropic support and the decision was made to go to the operating room and attempt to wean ECMO. Unexpectedly, when ECMO was stopped her blood pressure suddenly dropped to 43/35 and the end tidal CO_2_ was lost (<10 mm Hg) despite adequate ventilation. Transesophageal echocardiography was urgently performed demonstrating probable RVOT graft obstruction (Figure 2) with estimated right ventricular systolic pressure of >100 mm Hg (Figure 3). Epicardial imaging of the RVOT confirmed an obstruction (Figure 4). The obstructed graft was found to be thrombosed with thrombus extension to the main pulmonary artery up to the bifurcation, requiring graft replacement and pulmonary artery thrombectomy. The patient was weaned from CPB with high dose vasoactive support and inhaled nitric oxide, avoiding ECMO support due to the challenges faced with anticoagulation and bleeding. Despite this stormy course, she had a slow, steady recovery and continues to do well today.

## 2. Discussion

This case illustrates the known potential complication from Melody valve implantation with left coronary artery compression [1–4] despite optimal preventive measures, in which a novel rescue therapy the Impella 2.5 percutaneous LVAD was used as a bridge to definite surgical management and transport from the catheterization laboratory to the operating room. Previous reports have described CPR and cardiac massage to crush the valve until a definitive surgical solution could be achieved or coronary artery stenting [3, 4]. In this case an Impella LVAD placed via the femoral artery provided left ventricular unloading while supporting cardiac output during transport to the operating room. Another unique feature of this case is the development of RVOT and main pulmonary artery thrombosis in the setting of ECMO complicated by a severe bleed during which heparin was held and blood products were transfused. Bleeding and thrombosis are known complications of ECMO with clot in the ECMO circuit, intracardiac thrombus, microemboli, limb ischemia, and cerebral vascular accidents all being previously described [5–7]. A low flow state within the right ventricle and RVOT in addition to a decrease in anticoagulation left this patient vulnerable to this unexpected complication. Right heart and pulmonary graft thrombosis do not appear to have been previously described in this patient population.
